# Comparison of efficacy of needle-free injection versus injection by needle for iron supplementation of piglets: a double blind randomized controlled trial

**DOI:** 10.1186/s40813-022-00296-5

**Published:** 2023-01-16

**Authors:** T. J. Tobias, J. C. M. Vernooij, A. van Nes

**Affiliations:** Department of Population Health Sciences, Farm Animal Health Unit, Faculty of Veterinary Medicine, Utrecht University, Yalelaan 7, 3584CL Utrecht, The Netherlands

**Keywords:** Iron supplementation, Haemoglobin, Haematocrit, Anaemia, Growth, Swine, Welfare, Needle-free injection, RCT

## Abstract

**Background:**

In pig husbandry, most piglets receive an intramuscular injection with iron around three days of age for the prevention of hypochromic, microcytic anaemia. In recent years an increased interest is noted for needle-free injections, because of efficiency and safety for man and animal. This study aims to support the evidence on efficacy to extent the registration of a commercial iron supplement with a needle-free administration application. To this aim the study has two objectives: 1) to determine the effect of needle-free injection of the iron supplement on the mean blood Haemoglobin level at weaning, as primary outcome, and mean Haematocrit and mean Body weight of pigs at weaning as secondary outcome compared to no treatment, as main determinant of iron deficiency anaemia in piglets at time of weaning; 2) to compare the effects of needle-free administration of the iron supplement with regular injection by needle, with regard to the course over time of Haemoglobin, Haematocrit, piglet growth and the differentiated haematological and serum iron parameters.

**Methods:**

A double blind randomized controlled trial was conducted with 72 piglets, 8 piglets per litter from 9 litters. At three days of age pigs were selected, based on body weight, and random allocated to three study groups: a) control non-treated group (2 pigs per litter, 18 in total), b) a group with regular iron injection by needle injection (3 pigs per litter, 27 in total), c) a group that received iron by needle-free injection (3 pigs per litter, 27 in total). At four points in time (day 3, 14, 26 and 40) piglets were weighed and bled to analyse the dynamics of red blood counts and haematological parameters as well as serum iron parameters. The primary outcome parameter was the Haemoglobin (Hb) level on day 26. Of secondary importance were Haematocrit (Ht) and body weight (BW) at weaning and parameters with tertiary importance were the course of Hb, Ht and differentiated red blood cell parameters, serum iron, iron binding capacity and iron saturation. In the statistical analyses, linear mixed effect regression modelling was used to account for repeated measures within litters and pigs.

**Results:**

The analyses showed that needle-free administration was as efficacious to prevent iron deficiency anaemia at day 26 as administration using regular needle injection, compared to the control group. The mean level of Hb and Ht of pigs in the needle and needle-free group did not differ significantly. No side effects were observed.

**Conclusion:**

It is concluded that needle-free iron administration of the tested product is as efficacious as regular administration by needle injection.

**Supplementary Information:**

The online version contains supplementary material available at 10.1186/s40813-022-00296-5.

## Background

Iron is essential to enable haemoglobin to supply oxygen to body tissues. Piglets with an iron deficiency may develop a hypochromic microcytic anaemia in the first weeks of life. Piglets are very sensitive to develop iron deficiency anaemia for several reasons. First, iron is minimally transported from the sow to its offspring during gestation [1]. Therefore, piglets are born with a very low amount of iron reserve in the liver. Secondly, sow´s milk contains insufficient amount of iron (± 0,2 mg/100 ml) [2] to meet the piglets’ need (± 10 mg/day) (Zimmerman (1995) and von Bollwahn et al. (1983) as reviewed by Svoboda et al. [3]). Thirdly, piglets’ growth is extremely high compared to other animals, as they may increase in body weight by five times in the first four weeks of life. Although oral iron supplementation with specific products have shown to be effective to prevent anaemia in most of the weaned pigs, as reviewed by Svoboda and Píšťková [4], voluntary uptake of iron from the environment by pigs kept in modern husbandry under hygienic conditions usually seems insufficient to cover the iron needs of rapid growing piglets.

Clinically, pigs with iron deficiency anaemia are prone to growth retardation [5, 6], depression and their skin appears pale [7]. Recent literature suggests that piglets with iron deficiency not only develop an anaemia but are also considered to become cognitively impaired, for instance in spatial cognition [8, 9], but probably only when anaemia occurs pre-weaning [10]. Therefore, iron supplementation in early life seems to remain relevant for pig health and welfare.

Iron supplementation will result in fewer pigs with (sub)clinical signs of anaemia, such as low haemoglobin (Hb), low haematocrit (Ht) and low free serum iron [7, 11]. In addition, subclinical anaemic pigs may show high iron binding capacity (IBC) to serum transferrin, next to low iron saturation. In hypochromic anaemic pigs usually Mean Cell Volume (MCV), Mean Cell Haemoglobin (MCH), Mean Cell Haemoglobin Concentration (MCHC) and Cell Haemoglobin of Reticulocytes (CHr) are decreased, whereas Relative Distribution Width (RDW) is increased [7].

To infer on iron deficiency anaemia in the field, the clinician needs to consider many sources of information, rather than only a single Hb measurement. On the one hand, reference values for Hb, corrected for age, breed, growth potential and production phase, are scarce and often based on small studies under specific circumstances. For instance, some recent studies (e.g. [6, 12–14] use 5.59 mmol/L (= 9 g/L) as threshold for Hb, but these all indirectly refer to the minimal Hb value provided in a handbook [15] from data of nine piglets of twenty days of age from one litter housed under specific circumstances. The minimal value of such a dataset as threshold may not be considered adequate, as in human medicine it is suggested to refer to the lower limit value of the confidence interval for specific strata based on great population studies [16]. In contrast, other studies in pigs have used higher threshold values for Hb of 6.21 mmol/L or even 6.83 mmol/L, as reviewed by [3] to conclude on the occurrence of anaemia. On the other hand, even at much lower levels of below 4.96 mmol/L Hb (= 8 g/L) overt signs of anaemia may be absent [17]. To assess anaemia, erythropoiesis and iron availability in young pigs it is therefore recommended to use a combination of haematological as well as iron parameters [3]. For convenience, in this manuscript ‘iron deficiency anaemia’ is used for a state when the average Hb, MCV, MCH, MCHC, PCV or iron saturation is decreased, or when IBC and RDW is increased relative to measurements at an earlier time point or to the comparative group.

To prevent iron deficiency anaemia, in pig husbandry, piglets are commonly provided with iron in a gleptoferron or iron-dextran formulation by injection around three days of age. Iron supplementation by injection results in intramuscular or subcutaneous deposition of the product, conditional to the specifications of the needle, and the location and direction, or injection device that is used. Generally, injection of iron is considered to be efficient and efficacious [3, 13].

The use of needle-free-devices for injection of other pharmaceutical substances has gained popularity in both pig farming as well as in human medicine [18]. Needle-free injection of iron of pigs is advantageous from efficiency point of view as well as potentially reduced risk of microbial translocation and pathogen transmission by needles [18]. From an animal welfare point of view needle-free devices may be beneficial as well, but this aspect has not been studied to the knowledge of the authors. Particularly for novel administration routes, potential side-effects or the risk of iron toxicosis should be considered, characterized by neurological signs, such as tremors and ataxia, and mortality within few hours after administration [3]. In pigs with iron toxicosis, serum iron is expected to be elevated, transferrin binding capacity minimal and saturation extremely high.

The efficacy of needle-free iron injection to prevent iron deficiency anaemia has been limitedly documented [19, 20]. Therefore, this study has two objectives:

1. to determine the effect of needle-free injection of the iron supplement on the mean blood Haemoglobin level at weaning, as primary outcome, and mean Haematocrit and mean Body weight of pigs at weaning as secondary outcome parameters compared to no treatment, as main determinant of iron deficiency anaemia in piglets at time of weaning;
2. to compare the effects of needle-free administration of the iron supplement with regular injection using a needle, with regard to the course over time of Hb, Ht, piglet growth and the differentiated haematological and serum iron parameters, as tertiary outcomes.

## Results

### Descriptive data

A double blinded randomized controlled trial was conducted with 72 piglets in three study groups on one multiplier sow farm. Piglets were selected from nine litters, randomly assigned to a study group (two in group C and three in NF and N groups) on day 3 and followed up until forty days of age. Primary outcome parameter was the mean Hb at 26 days of age and of secondary importance are mean Hb and Ht and mean body weight. Tertiary outcomes were mean level and course of haematological and iron parameters and body weight over time.

On day 26, Hb and Ht of treated groups was higher than of the non-treated group C (Table 1). Body weight of the three groups was comparable around 7.8 kg. For a numeric summary of the results of the primary and secondary parameters and their course over time we refer to Tables 1 and 2, respectively.

**Table 1:** Mean and 95% confidence interval of primary outcome Haemoglobin (Hb), and secondary outcomes Haematocrit (Ht) and Body weight on day 26 of age per treatment group and estimated difference with 95% confidence intervals (95% CI) between Needle and Needle-Free treatment with Control group.

Study group	Mean	95% CI		Estimated difference	95% CI	
		Lower limit	Upper limit		Lower limit	Upper limit
Hb (mmol/L)						
Control	5.89	5.43	6.36	Ref^a^		
Needle	7.33	7.17	7.47	1.43	1.06	1.81
Needle-free	7.28	7.00	7.55	1.38	1.01	1.75
Ht (vol%)						
Control	0.32	0.30	0.34	Ref^a^		
Needle	0.38	0.37	0.39	0.06	0.04	0.08
Needle-free	0.37	0.36	0.39	0.05	0.03	0.07
Body weight (kg)						
Control (C)	7.86	7.13	8.58	Ref^a^		
Needle (N)	7.92	7.41	8.42	0.06	− 0.58	0.71
Needle-free (NF)	7.54	7.00	8.08	− 0.33	− 0.98	0.32

Iron supplement was administered at day 3

^a^Reference for comparison with other treatments

**Table 2:** Mean and 95% confidence interval (95% CI) of serum Haemoglobin, Haematocrit, body weight and iron parameters per day* per group**

Parameter	Study group**	Day 3	Day 14	Day 26	Day 40
Haemoglobin (mmol/L)	C	5.41 (5.10–5.72)	5.24 (4.78–5.70)	5.89 (5.43–6.36)	6.01 (5.78–6.24)
	N	5.25 (5.00–5.51)	6.73 (6.49–6.97)	7.33 (7.17–7.47)	6.47 (6.31–6.63)
	NF	4.95 (4.65–5.24)	6.64 (6.33–6.94)	7.28 (7.00–7.55)	6.38 (6.18–6.58)
Haematocrit (Vol%)	C	0.29 (0.27–0.30)	0.28 (0.26–0.31)	0.32 (0.30–0.34)	0.32 (0.31–0.34)
	N	0.28 (0.26–0.29)	0.35 (0.34–0.36)	0.38 (0.37–0.39)	0.34 (0.33–0.35)
	NF	0.26 (0.24–0.28)	0.35 (0.33–0.36)	0.37 (0.36–0.39)	0.33 (0.31–0.34)
Body weight (kg)	C	1.97 (1.81–2.12)	4.65 (4.25–5.04)	7.86 (7.13–8.58)	12.52 (11.54–13.50)
	N	1.92 (1.81–2.03)	4.56 (4.25–4.87)	7.92 (7.41–8.42)	12.59 (11.84–13.34)
	NF	1.91 (1.82–2.00)	4.26 (3.91–4.61)	7.54 (7.00–8.08)	12.26 (11.80–12.71)
Serum iron (µmol/L)	C	12.63 (9.23–16.04)	18.67 (10.40–26.93)	23.72 (15.37–32.08)	30.60 (25.49–35.72)
	N	13.77 (9.82–17.71)	37.97 (33.10–42.84)	27.05 (22.36–31.75)	27.57 (23.24–31.89)
	NF	14.87 (11.54–18.19)	34.13 (29.88–38.37)	29.21 (26.36–32.06)	26.12 (22.06–30.18)
Iron binding capacity (µmol/L)	C	51.09 (42.11–60.06)	86.81 (73.29–100.33)	92.73 (83.48–101.97)	79.14 (73.08–85.19)
	N	50.07 (41.25–58.89)	54.70 (48.16–61.24)	65.97 (59.23–72.71)	72.06 (68.25–75.88)
	NF	56.30 (48.72–63.88)	50.24 (44.93–55.56)	62.35 (57.70–67.00)	68.72 (65.57–71.87)
Iron saturation (%)	C	29.62 (18.49–40.75)	27.70 (13.13–42.26)	28.97 (17.00–40.94)	38.51 (31.85–45.18)
	N	37.47 (24.25–50.68)	70.52 (63.41–77.62)	44.80 (35.58–54.01)	38.46 (32.59–44.33)
	NF	31.56 (22.68–40.45)	69.20 (62.30–76.10)	47.93 (42.69–53.18)	37.91 (32.58–43.24)

Treatment with iron supplement administration started at day 3

^*^Days after birth

^**^C Control group, *N* Needle group, *NF* Needle-Free group

### Data analysis

Variation of the observed Hb and Ht values, in each study group were not similar at D26. In other words, the standard deviation of Hb in the Control group was about four times as large as the standard deviation in the needle group and twice as high compared to the needle-free group. In Ht values the ratio between standard deviations was 2.5 and 1.2 higher in the Control group compared to needle group and needle-free group respectively. The absolute increase in variation however was limited (results not shown).

### Primary outcome; Hb on day 26

**Fig. 1:**
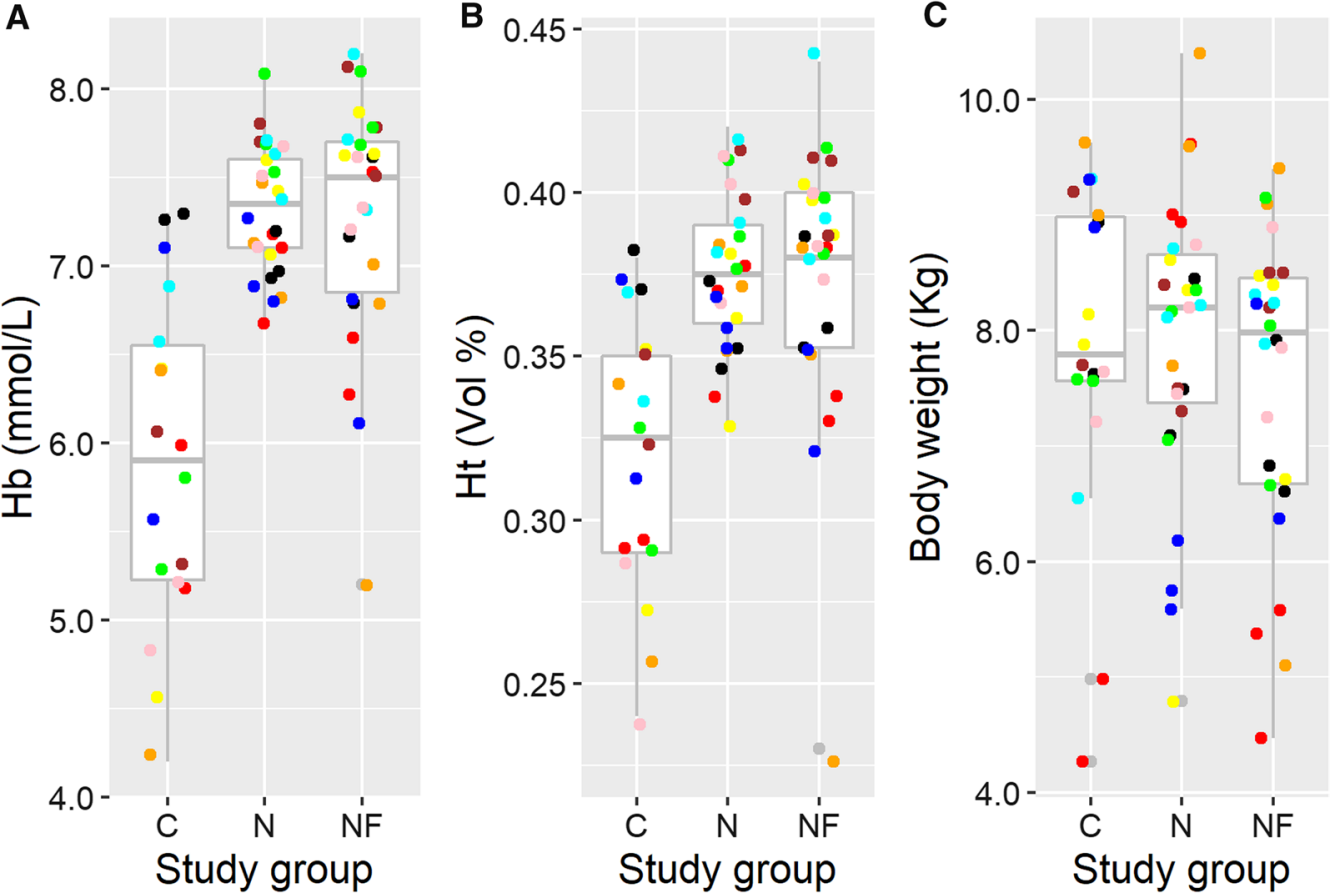
Boxplots of Haemoglobin (panel **A**), Haemotocrit (**B**) and Body Weight (**C**) of pigs at day 26 in the three study groups (*C* control, *N* Needle injection, *NF* needle-free injection). The different colours represent the origin from the nine different litters

The multivariate analysis showed that the mean Hb of pigs in both treated groups was significantly higher compared to mean Hb of pigs in group C (Fig. 1 and Table 1) on day 26. Additional analysis to determine the difference between Hb of group N and group NF using the NF as a reference group showed that the N pigs have an on average 0.07 mmol/L higher Hb (95% CI: −0.16–0.29), which was not significantly different.

### Secondary outcome parameters

#### Haematocrit and body weight on day 26

The multivariate analysis showed that the mean Ht of pigs in both treated groups was significantly higher compared to the mean Ht of pigs in the Control group (Fig. 1B and Table 5). Additional analysis to determine the difference between mean Ht of group N and group NF using the NF as a reference group showed that the N treated pigs have a similar mean Ht (estimate for the difference = 0.00, 95%C﻿I: −0.01 to 0.02).

**Fig. 2:**
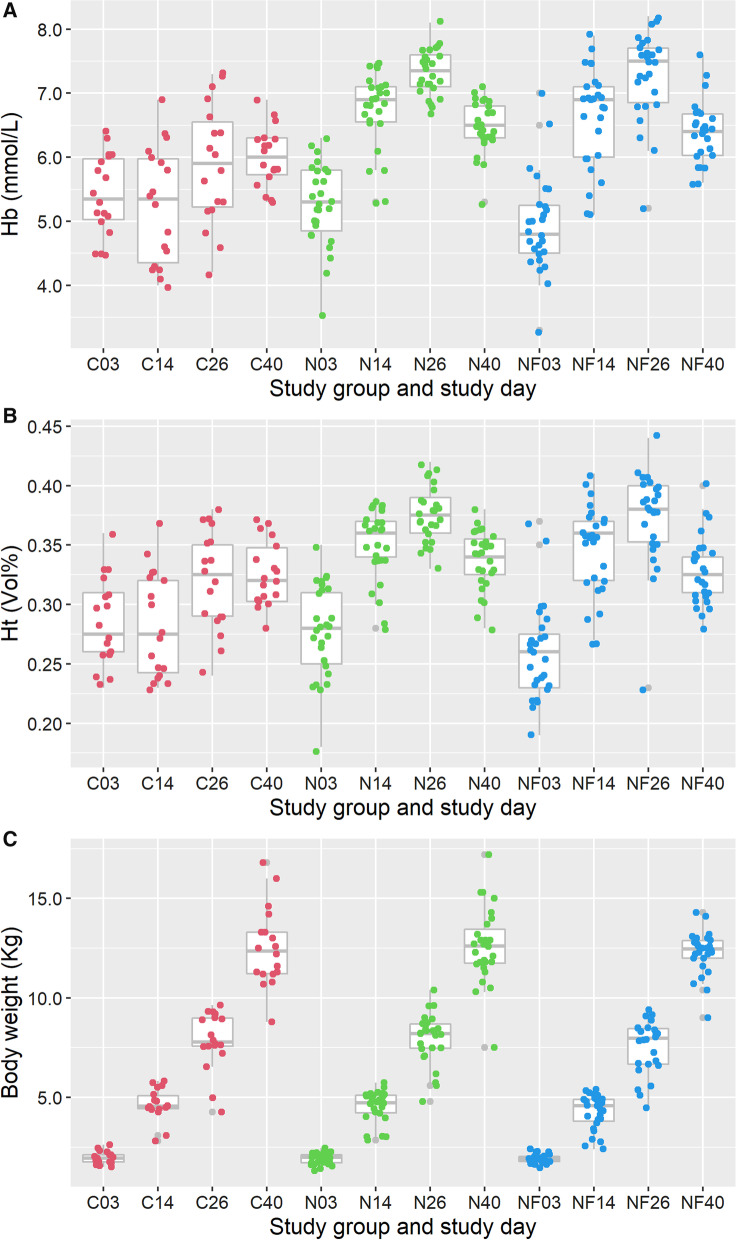
Course of Haemoglobin (in mmol/L) (panel **A**), Haematocrit (in Vol%) (panel **B**) and Body weight (in Kg) (panel **C**) over time per study group. C03 = the values of the Control group on day 3, etc. (*C* Control, *N* Needle and *NF* Needle-Free)

The average body weight of pigs on day 26 between study groups was not significantly different (Fig. 2C and Table 5).

### Tertiary outcome parameter results

Results of analysis of the Hb, Ht and Body weight over time and other haematological and iron parameters are presented graphically and briefly explained.

#### Hb in time

The statistical model for comparison of Hb over time contains group and time and interaction between group and time as explanatory variables (Table 3). Multivariate analysis of the course of Hb over time indicated that mean Hb in both treatment groups was significantly higher on day 14, day 26 as well as on day 40 compared to the C pigs (Fig. 2 and Table 3) but on day 40 the difference in means seemed to decline compared to the previous time points. The estimated mean Hb on day 3 in the needle-free group was significantly lower than the mean Hb in the needle injection and control group (Table 3). However, this difference was not observed as such at later points in time. Additional analysis on the dynamics of Hb of individual piglets showed that piglets with below-average Hb on day 3 generally remained to have a lower Hb compared to piglets with above-average Hb on day 3 (not shown). To consider exceptional circumstance number 4; piglets that were sampled twice on day 3, due to clotting in the first sample, did not show a significantly different Hb level on day 3 than piglets that were only sampled once (results not shown).

**Table 3:** Difference in means and 95% confidence interval (95% CI) between three iron supplementation methods (Needle-Free injection, injection by needle and no treatment) in course of time on the level of Hb (mmol/L), derived from the multivariate model

Model parameter	Time point	Effect estimate	95% CI	
		(mmol/L)	Lower limit	Upper limit
Mean Hb of control group	On day 3	5.41	5.10	5.72
Effect of time	Day 14	− 0.17	− 0.52	0.18
	Day 26	0.48^a^	0.13	0.83
	Day 40	0.60^a^	0.25	0.95
*Additional effects (difference between means) of group at time points relative to the same time point of the Control group*				
Needle group	On day 3	− 0.16	− 0.52	0.20
	Day 14	1.49^b^	1.13	1.85
	Day 26	1.43^b^	1.07	1.80
	Day 40	0.46^b^	0.10	0.83
Needle-free group	On day 3	− 0.46^c^	− 0.83	− 0.10
	Day 14	1.40^b^	1.03	1.76
	Day 26	1.38^b^	1.01	1.74
	Day 40	0.37^b^	0.00	0.73

If a confidence interval includes the 0 value there is no significant difference between group means. In this model significant effects of time (^a^) and group (^b^) are observed on Hb. ^c^The Needle-Free group had a slightly lower Hb on day3 compared to the Control group

#### Ht in time

The course of the Ht over time is graphically represented in Fig. 2 and numerically in Table 2. The model that fitted the data best to describe Ht was a model that contains group and time and the interaction between both as explanatory variables (Table 4). Multivariate analysis of the course of Ht over time indicated that mean Ht in both treated groups was significantly higher on day 14 and day 26 but not on day 40 (Fig. 2B and Table 4). In addition, the Ht of pigs from group NF shows a greater variation than that of pigs from group N, but the absolute difference in variation was rather small. As with the Hb parameter, also the mean Ht of pigs from the NF group was significantly lower than that of pigs from group N on day 3 only (Table 4). Additional analyses showed that Ht of individual piglets with lower Ht on day 3 remained on average slightly lower compared to the mean Ht of pigs with an above-average Ht on day 3 (results not shown), but this did not seem to affect mean Ht on later points in time (Table 4).

**Table 4:** Estimated difference in means and the 95% confidence interval* of Haematocrit (Ht in vol%) between three iron supplementation methods (Needle-Free injection, injection by needle and no treatment) in course of time from the multivariate model

Model parameter	Time point	Effect estimate (Vol%)	95% confidence interval	
			Lower limit	Upper limit
Mean Ht of C group	On day 3	0.29	0.27	0.30
Effect of time	Day 14	0.00	− 0.02	0.02
	Day 26	0.03	0.01	0.06
	Day 40	0.04	0.02	0.06
*Additional effects (difference between means) of group at time points relative to the same time point of the Control group*				
Needle group (N)	On day 3	− 0.01	− 0.03	0.01
	Day 14	0.07^b^	0.05	0.09
	Day 26	0.06^b^	0.04	0.08
	Day 40	0.01	− 0.01	0.03
Needle-free group (NF)	On day 3	− 0.03^a^	− 0.04	− 0.01
	Day 14	0.06^b^	0.04	0.08
	Day 26	0.05^b^	0.03	0.07
	Day 40	0.00	− 0.02	0.02

*If a confidence interval includes the 0 value there is no significant effect observed

^a^A significant difference in the NF group is observed on day 3 compared to group C

^b^Significant increases of Ht on day 14 and 26 in group N and NF are observed as well

#### Body weight in time

The average body weight of pigs on day 3 did not differ between the groups either, which indicates that randomization and allocation to study groups of the piglets was executed accordant to the expectations. In addition, no significant difference was observed between body weight of pigs in the three study groups at other points in time (day 14 and day 40) (Table 5).

**Table 5:** Estimated difference in means and 95% confidence interval (95% CI) between three iron supplementation methods (Needle-Free injection, injection by needle and no treatment) in course of time on the body weight (in Kg), derived from the multivariate model

Model parameter	Time point	Effect estimate (Kg)	95% CI	
			Lower limit	Upper limit
Mean weight group C	Day 3	1.97	1.42	2.52
Effect of time	Day 14	2.68^a^	1.97	3.39
	Day 26	5.89^a^	5.18	6.60
	Day 40	10.56^a^	9.85	11.26
*Additional effects (difference between means) of group at time points relative to the same time point of the Control group*				
Needle group	Day 3	− 0.05	− 0.69	0.60
	Day 14	− 0.09	− 0.73	0.56
	Day 26	0.06	− 0.58	0.71
	Day 40	0.07	− 0.58	0.72
Needle-free group	Day 3	− 0.05	− 0.70	0.59
	Day 14	− 0.38	− 1.03	0.26
	Day 26	− 0.33	− 0.98	0.32
	Day 40	− 0.28	− 0.93	0.37

If a confidence interval includes the 0 value there is no significant effect observed

^a^In this model only significant effects of time on body weight are observed but not between groups

#### Iron associated parameters

##### Serum iron concentration

**Fig. 3:**
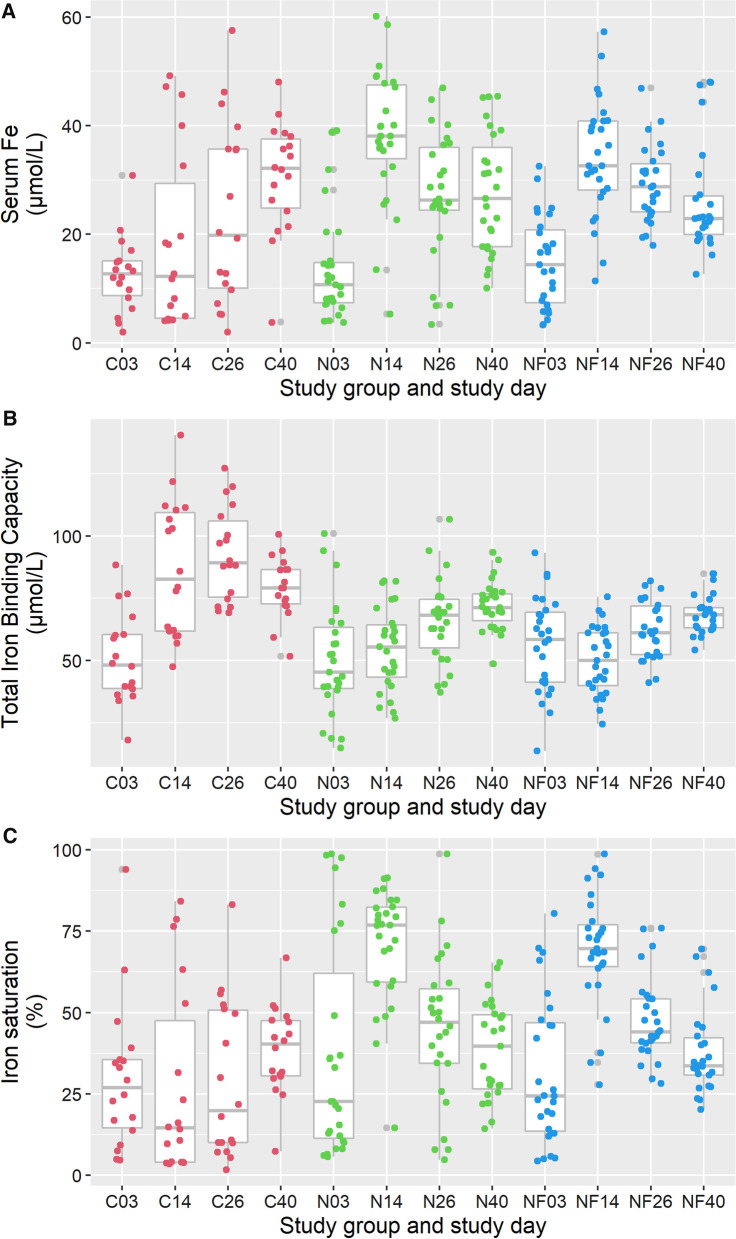
Boxplots of the course of serum iron (Fe in µmol/L) (panel **A**), Total Iron Binding Capacity ((µmol/L) (panel **B**) and Iron saturation (%) (panel **C**) in time per study group. C03 = the values of the Control group on day 3, etc. (*C* Control, *N* Needle and *NF* Needle-Free). Dots represent the values of individual pigs

Mean serum iron (Fe) increased at all time points in the control group (Fig. 3A). The serum Fe level in the treatment group showed a large increase to day 14 but dropped to a lower stable level afterwards. Compared to the control group both treatment groups (N + NF) showed a larger increase on Day 14 (Table 6). After day 14, in both treatment groups (N + NF) the mean Fe declined, compared to the mean Fe in the control pigs that inclines. Additional analysis showed that mean serum Fe on day 14 did not significantly differ between pigs from groups N and NF (results not shown).

**Table 6:** Estimated difference between mean at indicated day and mean at day 3 in Control for serum iron (Fe in µmol/L) with lower and upper limits of the 95% confidence interval (95%CI) between three iron supplementation methods (Needle-Free injection, injection by needle and no treatment) in course of time from the multivariate model

Model parameter	Time point	Effect estimate (µmol/L)	95% CI	
			Lower limit	Upper limit
Mean Fe of C group	on day 3	12.64	8.59	16.69
Effect of time	Day 14	6.03	− 0.82	12.89
	Day 26	11.08	4.61	17.56
	Day 40	17.97	11.97	23.97
*Additional effects of group at time points relative to the same time point of the control group*				
Needle group	On day 3	1.14	− 3.87	6.14
	Day 14	19.30	11.84	26.76
	Day 26	3.25	− 3.68	10.18
	Day 40	− 3.04	− 9.15	3.08
Needle-free group	On day 3	2.23	− 2.78	7.24
	Day 14	15.45	7.99	22.91
	Day 26	5.45	− 1.48	12.38
	Day 40	− 4.52	− 10.68	1.64

##### *Iron binding capacity (IBC)*

In the control group the IBC increased more compared to that of pigs from the treatment groups (Fig. 3B). Additional analysis showed that between groups N and NF there is no difference observed of the mean iron binding capacity on day 14, day 26 nor on day 40 (results not shown).

##### Iron saturation

In pigs from the treated groups (N + NF) the mean iron saturation was high on day 14 and has decreased thereafter (Fig. 3C). In pigs from the control group iron saturation was low and highly variable, but increases slowly after weaning (day 40). On day 14 there was no significant difference in iron saturation between the pigs in the N and NF groups. On day 40, the mean iron saturation was not significantly different between all three study groups (results not shown).

#### Hematological parameters

##### Mean red cell volume (MCV)

**Fig. 4:**
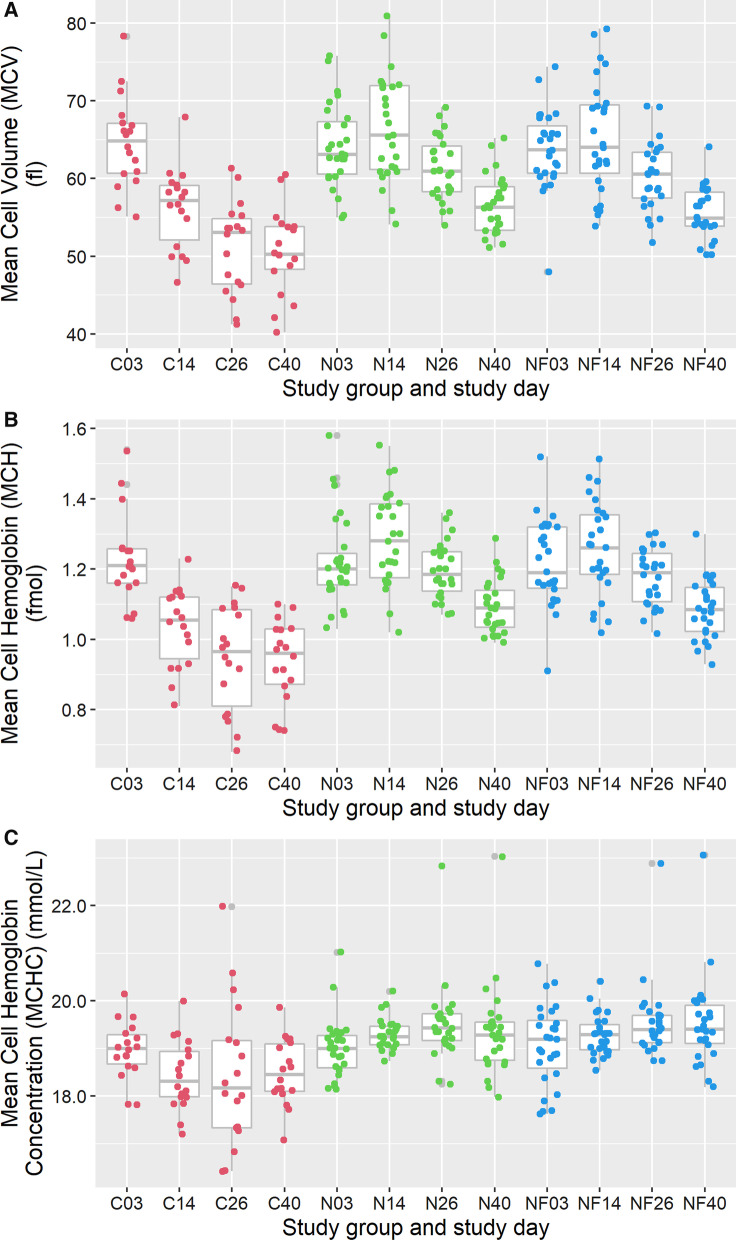
Course of Mean Cell Volume (in femtolitre) (panel **A**), Mean Cell Haemoglobin (in femtomole) (panel **B**) and Mean Cell Haemoglobin Concentration (MCHC) (in mmol/L) (panel **C**) over time per study group. C03 = the values of the Control group on day 3, etc. The boxplots represent the summary of values of individual pigs represented in coloured dots

The mean MCV of the control group decreased after day 3, whereas the mean MCV of the treatment groups was considered normal and similar on day 14 compared to day 3 and decreased only thereafter but remained at a higher level than the control group (Fig. 4).

##### Mean cell haemoglobin

The mean cell Haemoglobin (MCH) was higher in the treated animals, group N and NF, compared to the pigs in the control group on day 14. A lower MCH indicates that erythrocytes become hypochromic. In non-treated control animals mean MCH was already lower on day 14 and decreases even further. Mean MCH of treated animals decreases after day 14, but remained higher compared to MCH of the control pigs (Fig. 4).

##### Mean cell haemoglobin concentration

The Mean Cell Haemoglobin Concentration (MCHC) is the result of MCH and MCV. The non-treated control group had a lower mean MCHC after day 3 onwards compared to the other groups (Fig. 4).

##### Relative distribution width of erythrocytes (RDW)

**Fig. 5:**
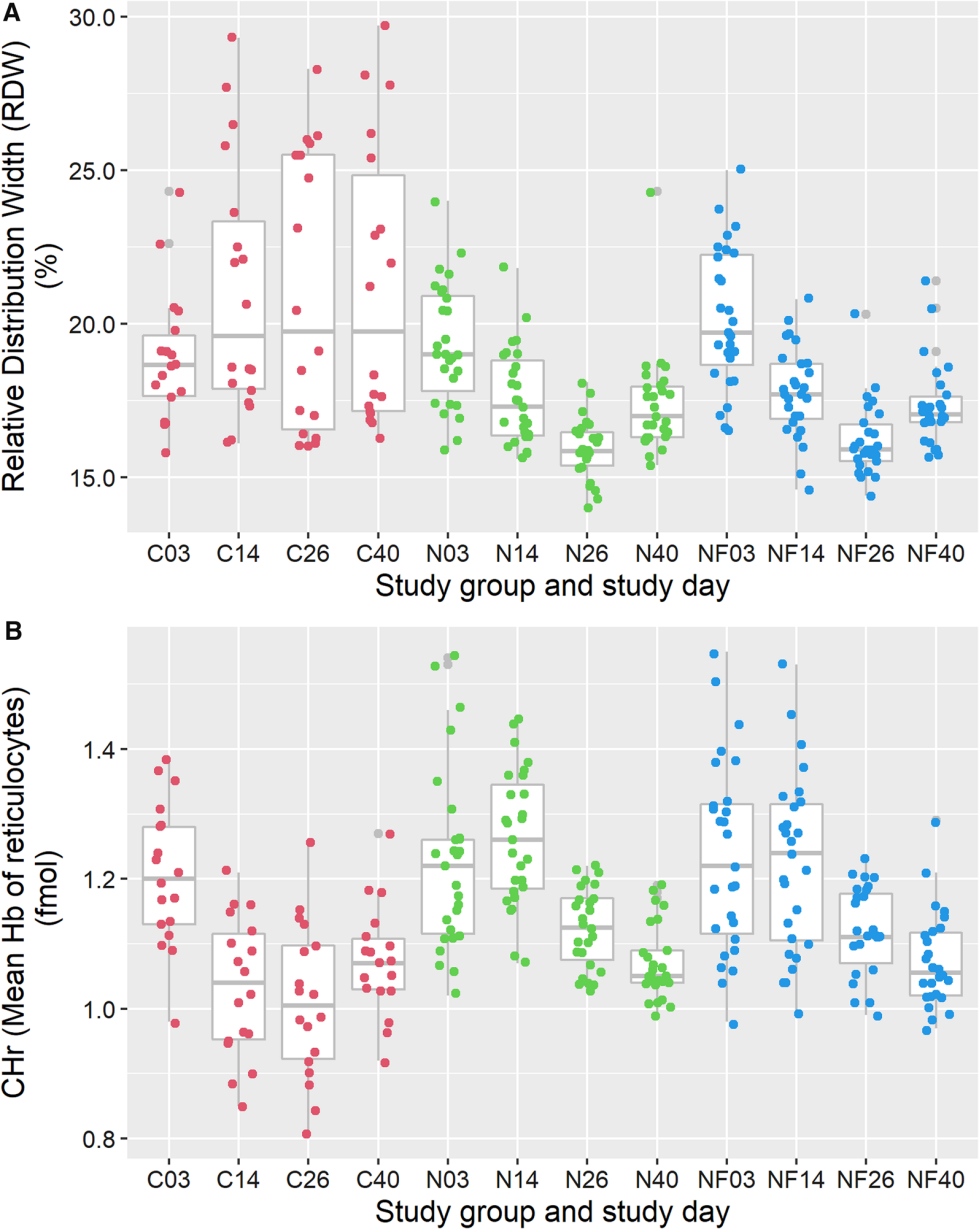
Course of Relative Distribution Width (RDW) (in %) (panel **A**) and mean Cell Haemoglobin of reticulocytes (CHr) (in femtomole) (panel **B**) in time per study group. C03 = the values of the Control group on day 3, etc. The boxplots represent the summary of values of individual pigs represented in coloured dots

The variation in size of erythrocytes (RDW) was highest in the untreated control group, which fits with an iron deficiency anaemia (Fig. 5). In other words, from day 14 in the control group erythrocytes were observed of many different sizes (very small to normal). Between groups N and NF no difference was observed in mean RDW.

#### Mean cell haemoglobin of reticulocytes (CHr)

In pigs of the control group, CHr decreased after day 3 and in the treated groups it decreased after day 14 (Fig. 5B). This matches with deficiency of available iron, as the serum iron concentration only increased after day 14 in the C group and already after day 3 in the NF and N groups (Fig. 3A). On day 40, CHr of all groups were more or less comparable (Fig. 5B).

### Side effects and survival

During the study no side effects regarding the administration of the iron supplement were observed. Although one pig of the needle-free group did not survive, this was considered not to be affected by the treatment, as the sow had crushed the pig on day 26. The pig was submitted for post mortem examination anyhow and additionally hemosiderosis was diagnosed; i.e. a deposition of hemosiderin, which is an iron containing pigment, in the lymph nodes, liver and kidneys. These depositions are more often observed in pigs of that age irrespective of the administration route of iron.

## Discussion

The aim of this study was to evaluate whether needle-free (NF) application of a commercial iron supplement was efficacious to prevent an iron deficiency anaemia at time of weaning (day 26). In fact, pigs in the control group showed to be anaemic, based on both Hb and Ht levels. The results of this study show that Hb as well as the Ht of the NF group was significantly higher compared to Hb and Ht of the non-treated group C. In addition it showed that Hb and Ht of the N and NF groups were very similar at time of weaning. These conclusions also hold statistically when the arbitrary cut-off value of 5.59 mmol/L Hb [15] is considered (analyses not shown). The Hb values of the pigs in the N and NF groups at time of weaning were all, except one in the NF group, above the referred threshold value for 20 day old pigs of 5.59 mmol/L Hb (= 9.0 g/dL) [15], whereas seven of the pigs in the control group had Hb values below 5.59 mmol/L.

In addition, the results of the iron saturation parameter indicated that the levels of iron saturation between the needle and needle-free group were not significantly different. Thereby we conclude that the risk of iron intoxication is not increased by the needle-free application compared to injection by needle. Moreover, it can be concluded that needle-free application of the product is efficacious to prevent reduced Hb levels at time of weaning (day 26). In addition, NF application of the product was as efficacious as regular injection by needle to prevent reduced Hb and Ht levels at weaning.

The variation of Hb and of Ht values at D26 between the study groups were unequal which made it necessary to account for this difference in the statistical analyses. Although the magnitude of the difference in standard deviation of Hb was highest in the control group and lowest in the needle group the absolute difference in standard deviation was limited. The difference was approximately 0.1 mmol/L for Hb, similar to what was observed by Kievit et al. [19] who studied efficacy of needle-free iron dextran supplementation in a similarly designed clinical trial. Although many explanations may be considered, the clinical relevance of the increased variation in Hb seems limited in this study. As a matter of fact, there was no significant difference in mean Hb and mean Ht between groups N and NF. However, the study was conducted with pigs with similar body weight, near the median weight within the litter. It could be hypothesized that in pigs of other sizes (especially the much heavier piglets with higher growth) an effect may be more pronounced, as suggested by Perri et al. [6]. However, we still consider the clinical relevance of higher variation of Hb in the NF group as minor as in this study neither effects on growth were observed nor signs of anaemia were seen.

Considering the prevention of iron deficiency anaemia, the effect of iron administration in both treated groups is sufficient to prevent the occurrence of hypochromic and microcytic (Fig. 4) anaemia as was observed in the control group from day 3 onwards. Also the decrease of MCV in the control group is indicative of microcytic anaemia (Fig. 4). When variation of erythrocyte size (RDW) and Hb level of reticulocytes (CHr) are considered, and given that these parameters do not differ between the pigs in groups N and NF (Fig. 5), it is concluded that erythropoiesis in both treated groups seem not to differ significantly. However, as this study was not designed to formally test numeric differences in RDW and CHr, formal conclusions cannot be drawn to this aspect.

In contrast to the expectations, body weight of pigs in all study groups was not significantly different and thus did not seem to be affected by iron administration in this study. In general, and to previous experience (e.g. [17, 19] iron administration prevents a decrease in growth due to iron deficiency anaemia. We cannot fully explain the observed result of no difference in mean weight between respective study groups in this study. One the one hand, one could conclude that severity of iron deficiency in the control group was low, as previously it has been suggested that growth retardation only occurs in severe cases of iron deficiency anaemia and just three control pigs had a Hb value below the presumed clinically relevant threshold value of 4.96 mmol/L at weaning. On the other hand it may be that subclinical anaemia had already peaked in control pigs well before weaning, as on day 14 eight pigs had an Hb below 4.96 mmol/L.

Effects of iron deficiency may be more pronounced at higher age and in case iron intake from other sources, e.g. creep feed and coprophagy, is insufficient. In this farm, feed intake before and after weaning is known to be relatively high, as also reflected by the growth rate of weaned pigs, and this could have resulted in relatively high intake of iron even in the control group.

Although effects of lack of iron in the Control group until weaning can be observed by many haematological analyses (Figs. 3, 4 and 5), at D40 differences between groups are smaller, likely due to more comparable intake of iron by feed intake. Still, based on RDW at D40 one can conclude that anisocytosis is more relevant in the control group, reflecting suboptimal erythropoiesis at an earlier point in time, whereas the CHr results of D40 suggest that erythropoiesis in all groups is more comparable at that time.

Despite that mean body weights on day 3 were not significantly different between study groups, the average Ht of pigs in group NF was significantly lower than of the C and N groups. As, some pigs were sampled twice on day 3, due to clotting in the first sample, the Ht and Hb of samples from these samples was evaluated and found not to be different compared to those samples that were obtained from pigs that were bled only once. Therefore, the effect of sampling twice on the difference of Hb is considered irrelevant. The lower Hb on day 3 is therefore considered a coincidence for which a specific cause or explanation is not available. As the effect of a lower Hb in the NF group on day 3 was not observed on day 14, the difference of Hb on day 3 is also considered as irrelevant.

Finally, the IBC between groups N and NF were not significantly different, neither was iron saturation or serum iron on day 14. Together with the absence of clinical signs, the “normal” serum iron concentration, this indicates that the availability and binding of iron in both treated groups was similar.

So, although clinical signs of anaemia nor iron toxicosis were apparent, iron supplementation significantly affected haemoglobin and other iron and haematological parameter values. Based on these results, one may still hypothesize that providing iron to pigs with high growth or high feed intake could be further optimized regarding time, dosing and routes, to save costs and improve animal welfare. The results suggest that such iron deficiency anaemia prevention plans may need to be farm specific.

## Conclusions

Needle-free injection of the commercial iron supplement is efficacious to prevent an iron deficiency anaemia at time of weaning (day 26). Needle-free injection of the commercial iron supplement was as efficacious to prevent iron deficiency anaemia at day 26 as administration using regular needle injection, compared to the control group. The level of Hb and Ht of pigs in the needle and needle-free group did not differ significantly either.

The conclusion of this study is that efficacy and safety of needle-free injection the commercial iron supplement product did not differ from the efficacy and safety of regular injection by needle to prevent iron anaemia deficiency.

## Methods

De study had two objectives:

1. to determine the effect of needle-free injection of the iron supplement on the mean blood Haemoglobin level at weaning, as primary outcome, and mean Haematocrit and mean Body weight of pigs at weaning as secondary outcome parameters compared to no treatment, as main determinant of iron deficiency anaemia in piglets at time of weaning;
2. to compare the effects of needle-free administration of the iron supplement with regular injection by needle, with regard to the course over time of Hb, Ht, piglet growth and the differentiated haematological and serum iron parameters, as tertiary outcomes.

The primary study outcome was defined as the level of Hb at weaning (day 26) and secondary outcome parameters at weaning were the Ht and body weight at weaning. Outcomes of tertiary interest were the differentiated haematological and iron parameters and piglet growth and their course over time.

### Study location

The study has been conducted in 2017 at Utrecht University, Faculty of Veterinary Medicine at the ‘Academic Training Facility’ “De Tolakker”. “De Tolakker” is a conventional sow farm with at time of study 190 sows (Finnish Landrace x York) with an average to above-average level of production considering the number of weaned piglets per sow per year (32 weaned pigs/sow/year and 16.6 life born pigs/litter). Farrowing sows are housed in a pen with a farrowing crate. The piglet nest floor is covered with some saw dust. Creep feed is provided ad libitum from day 7 onwards, containing on average 220 mg/Kg iron. Sow faeces is removed daily and access to sow’s feed is limited.

### Study design

A double blind randomized controlled trial (RCT) was designed, adjusted from a previous study [19]. The bio-statistician, as well as the blood sampler and the animal care takers were blinded to the study group status of the individual pigs, and the laboratory results until after concluding the statistical analyses.

Seventy-two (72) piglets, from nine litters, were randomly allocated to three study groups:

(A). 27 piglets for the needle-free administration group (NF), three piglets per litter,
(B). 27 piglets in the group with regular injection by needle (N), three piglets per litter,
(C). 18 piglets in a non-treated control group (C), two piglets per litter.

T﻿he study started when all piglets were minimal 3 days old and maximum 5 days old (further referred to as day 3). The pigs that were included in the study were selected based on body weight on day 3; i.e. the eight pigs that had a body weight around the within litter median body weight were included in the study and randomly allocated to one of the three study groups. Other piglets in the nine litters remained with the sow and were treated with iron by injection, but were excluded from the study and follow-up. In case of excess litter size at birth, compared to the number of mammary glands, excess piglets were cross fostered two days before start of the study, resulting in a median litter size of 14 (range 11–14).

Piglets were weighed and blood was collected on day 3, 14, 26 and 40. Blood sampling was performed before administration of the iron supplement in the afternoon on day 3 for the needle group (N) and the needle-free group (NF). Blood samples were obtained by gently fixing the pig in dorsal recumbency and obtaining blood from the *V. jugularis* or the *V. cava cranialis*. Samples were collected using 2.7 mL K3E S-Monovette® 66 × 11 mm tubes (EDTA) (Sarstedt, Nürnbrecht, Germany) and 2.7 mL S-Monovette® tubes with clot activator (Serum) (Sarstedt, Nürnbrecht, Germany), in combination with Safety-Needle 22Gx1½” (Sarstedt, Nürnbrecht, Germany). For samples of only day 3 it appeared that 10 of 72 samples contained too much clotting for appropriate laboratory sample analysis. Therefore, these piglets were sampled again before iron injection later that day. This concerned the following number of animals from the following groups: C-group 1x, NF group 3x, N group 6x. Students’ t-test analysis showed that piglets, that were sampled twice on day 3, did not differ in Hb and Ht compared to piglets sampled only once on day 3 (p > 0.6) (results not shown). Therefore the results of the second sample were merged with those from piglets that were sampled once, for further statistical analysis.

Administration of 1 ml MS Ferrosafe (VIC-Animal Health B.V., Nistelrode, the Netherlands, RegNL 115374), containing 200 mg Fe^3+^, as gleptoferron (532.6 mg) per mL, to the N group was performed using a repetitive injection device for animals (Henke-Sass Wolf GmbH (Germany)) and a 21G × 5/8″ needle (Terumo Europe BV, Leuven, Belgium).

Transdermal administration via needle-free injection of 1 ml of the iron supplement to the NF group was performed using the MS Pulse (hereafter referred to as needle-free (NF)) (Bladel, the Netherlands). The NF system was calibrated on the day of administration to guarantee the administration of 1 mL per animal.

### Study size

Power calculations were performed for both research objectives on the primary outcome parameter Hb at day of weaning (D26). Assumptions for the calculations were derived from Kievit et al. [19], whom conducted a similar study but with a different iron supplement product. Non treated pigs will have a lower Hb, of at least 1 mmol/L less and about a factor 3 higher standard deviation of Hb in the control group compared to the iron supplemented group at day 26.

For the first objective (efficacy to prevent iron deficiency by NF iron administration), a minimum number of 15 piglets per group was necessary to detect with Power 80% and 5% significance level that there would be a true difference in Hb of more than 1.0 mmol Hb/ml between treated and non-treated pigs at weaning (day 26), considering one sided testing with a standard deviation of 0.471 and a ratio of the standard deviation between groups of 3.

For the second objective, comparison of NF and N iron administration, two sided power calculations were performed with results from Kievit et al. [19] (mean Hb = 7.1 (NF), Hb = 7.44 (N) and sd = 0.47 with a 1.24 ratio of sd for the NF compared to the N group). A minimum of 26 pigs for both treatment groups was needed to detect the same difference of 0.34 with 80% power at the 5% significance level (Type I error).

To control for expected loss to follow up due to mortality of pigs or potential disease of the sow and to allow equal numbers of piglets per litter for each study group the number of pigs was raised to 18 pigs for the control group and 27 pigs for both treatment groups (totalling to 72); stratified over nine different litters.

### Laboratory analyses

Samples were analysed on the same day as they were collected.

The laboratory analyses were conducted at the University Veterinary Diagnostic Laboratory (UVDL). Haematological analyses were conducted on an Advia 2120i analyser (Siemens, Germany) and the iron parameters were determined using an AU-680 Chemistry analyser (BeckmanCoulter, The Netherlands). The following parameters were obtained:

- Serum Haemoglobin (Hb) in mmol/L
- Haematocrit (Ht) (Vol%/100)
- Serum iron (Fe) in µmol/L
- Iron binding capacity (IBC) in µmol/L
- Iron saturation in %
- Total number of erythrocytes per L blood in × 10^12/L
- Mean cell volume (MCV) in fl (femtoliter)
- Mean cell Haemoglobin (MCH) in fmol
- Mean cell Haemoglobin concentration (MCHC) in mmol/L
- Relative Distribution Width (RDW), as a measure for variation of red blood cell volume in % (RDW = (standard deviation of MCV / mean MCV) × 100%.)
- Absolute number of reticulocytes in 10^9/L
- Relative number of reticulocytes in %
- Mean Haemoglobin concentration in reticulocytes (CHr) in fmol

### Statistical analyses

For analysis of the effects of needle-free injection compared to regular needle injection and compared to the non-treatment on Hb, Ht and Body weight a linear mixed effect model was used [21, 22]. This model takes repeated measurements per piglet within litter (random intercept) into account. In addition, it deemed necessary to adjust the model for unequal variances in Hb and Ht between treatment groups to compare the group means at D26. The effect of time (as factor), group and the interaction between both on the respective outcome variables was analysed using similar models but no adjustment for unequal variances was needed. To select the best but parsimonious statistical model, fit of the model to the data was evaluated using AIC (Akaike Information Criterion) [23]. In short, models with a lower AIC fit the data better and are preferred to models with higher AIC. Results are presented as estimated means or differences between means with 95% confidence intervals.

### Clinical follow up

All animals were inspected twice daily and received appropriate care in case of signs of disease. During the early phases of the trial signs of swelling at the injection site and potential effects due to iron intoxication, such as respiratory distress, weakness, ataxia, tremors and mortality were monitored clinically. In case of mortality, unexpected disease or side effects further clinical and/or post mortem examination would be performed.

During the study three minor events occurred and required intervention that need to be addressed. First, in the study plan all pigs included in the study would remain with their own mother sow during the suckling stage of the study. Unfortunately, one sow appeared too sick and lost too much body condition for which she had to be separated from her piglets immediately at day 5. The sow was replaced by a foster sow and so the entire litter, including the treated and untreated pigs were fostered by the new sow and remained in the trial. The results of the pigs (body weight and haematological analyses) did not show to be affected. Second, one pig from the needle-free group was crushed on day 26, just before the pig would have been weighted and blood would have been collected. Therefore, for this pig only the observations on day 3 and day 14 are included in the analyses. Finally, at the end of the study it became clear that one pig from the needle group was not sampled on day 26 as well as on day 40. Subsequently, blood was collected from this pig on day 41. In the analyses, it was assumed that the results on day 41 would be similar for this particular pig as it would have been when it was sampled on day 40.

## Supplementary Information

**Additional file 1: Data file**. The complete dataset for this study, available as spreadsheet file. The file contains the data (1. data sheet) and a legend (2. legend) that provides an overview of the abbreviated column headings in the data sheet and the measuring units. The data sheet is provided as is and contains the results from the piglets collected in the trial per study day and study group (body weight, piglets sex, litter of origin, haematological analyses and iron associated parameters supplemented). Additional output of the analyzer that was not used in this study is also included for use by others. Attribution of the authors upon use of the data is appreciated.

## Data Availability

The data that support the findings of this study can be found in a *csv*-formatted file as Additional file 1 to this manuscript.

## Abbreviations

C: Control study group that received no iron supplementation
Hb: Serum haemoglobin in mmol/L
Ht: Haematocrit in Vol%/100
Fe: Serum iron (Fe) in µmol/L
IBC: Iron binding capacity in µmol/L
MCV: Mean cell volume in fl (femtoliter)
MCH: Mean cell haemoglobin in fmol (femtomole)
MCHC: Mean cell haemoglobin concentration in mmol/L
N: Study group that received the iron supplement by Needle injection
NF: Study group that received the iron supplement by Needle-free injection
RDW: Relative distribution width (RDW), as a measure for variation of red blood cell volume in % (RDW = (standard deviation of MCV/mean MCV) × 100%)
CHr: Mean haemoglobin concentration in reticulocytes in fmol
